# Efficacy of a latency- and productive infection-deficient Gammaherpesvirus as a vaccine strategy

**DOI:** 10.1186/1750-9378-7-S1-O7

**Published:** 2012-04-19

**Authors:** Yong Hoon Kim, Leming Tong, Tiffany Hsu, Evan Shih, Ronika Sitapara Leang, Seungmin Hwang, Ren Sun, Ting-Ting Wu

**Affiliations:** 1 Department of Molecular and Medical Pharmacology, School of Medicine, University of California at Los Angeles, Los Angeles, CA, USA; 2 Dental Research Institute, School of Dentistry, University of California at Los Angeles, Los Angeles, CA, USA

## Background

Human gamma-herpesviruses, Epstein-Barr virus (EBV or HHV-4) and Kaposi’s sarcoma-associated herpesvirus (KSHV or HHV-8), are associated with several malignancies, especially in AIDS patients. Although highly active antiretroviral therapy (HARRT) has significantly reduced the incidence of EBV- and KSHV-associated tumors, it does not eliminate EBV or KSHV infection, and the tumor risk remains high for HIV-1-infected individuals who are also carriers for EBV and KSHV. Tumorigenesis of gamma-herpesviruses is associated with the persistence of infection. Thus, vaccination to elicit protective immunity that inhibits the establishment of viral persistence will prevent the occurrence of virus-associated cancers. However, currently there are no effective vaccines available for KSHV or EBV.

## Material and methods

For proof of concept vaccination experiments, we utilize a mouse gamma-herpesvirus infection model. Previously, we have shown that vaccination with a non-persistent highly lytic live attenuated virus (AC-RTA) provides effective protection against a challenge infection by the wild type virus. To increase the safety of vaccination, the *in vivo* lytic replication capacity of a live gamma-herpesvirus needs to be significantly weakened without losing immunogenicity. We hypothesize that removal of the viral genes that inhibit the host immune responses will reduce the fitness of the virus but potentially increase its immunogenicity. For this purpose, we have removed immune evasion genes from AC-RTA along with other modifications.

## Results

The resultant virus named DIP (**D**eficient in **I**mmune evasion and **P**ersistence), replicates in cell culture but is severely attenuated in mice, deficient in acute productive infection and latency. However, immunization of the DIP virus prevents latency establishment by the challenge wild type virus. Next, we aim to test strategies to improve the immunogenicity in immunocompetent and CD4-deficient hosts by incorporating expression of co-stimulatory molecules into the DIP virus.

## Conclusions

The non-persistent DIP virus that undergoes limited *in vivo* viral replication provides us a novel vaccine strategy for preventing infection of human gammaherpesviruses. The “proof of concept” study in the mouse infection model is necessary to provide fundamental insights into the development of vaccines for the tumor-associated human herpesviruses.

